# Sinonasal mucosal melanoma: treatment strategies and survival rates for a rare disease entity: A single center experience and review of literature

**DOI:** 10.1007/s00508-021-01847-6

**Published:** 2021-04-12

**Authors:** Alexandros Andrianakis, Peter Kiss, Markus Pomberger, Axel Wolf, Dietmar Thurnher, Peter Valentin Tomazic

**Affiliations:** Department of Otorhinolaryngology, Head and Neck Surgery, Medical University of Graz, Auenbruggerplatz 26, 8036 Graz, Austria

**Keywords:** Nasal mucosa, Paranasal sinuses, Nasal cavity

## Abstract

**Background:**

Sinonasal mucosal melanoma (SNMM) is a rare disease entity comprising 0.4–1.3% of all melanomas. Surgery with free margins has been the primary treatment over decades. Neither the addition of radiotherapy nor chemotherapy could significantly improve outcome rates of this devastating malignancy. This study presents our clinical experience with SNMM over a 19-year period and summarizes the current body of literature on SNMM.

**Methods:**

This retrospective analysis included 12 patients with SNMM treated from 2001 to 2019 at an academic center. Additionally, a literature review of the last 29 years on treatment and survival data of SNMM was conducted.

**Results:**

Main initial symptoms were epistaxis and nasal obstruction. Of the patients 9 underwent endoscopic surgery, 6 received adjuvant therapy. 3 patients who did not undergo surgery, received chemoradiotherapy, radiotherapy alone, and chemotherapy alone, respectively. At the time of diagnosis 2 patients had distant metastases and 4 patients developed distant metastases during the course of the disease. Mean overall survival (OS) was 30.6 months, 3‑year and 5‑year OS were 25%, and 18.2%, respectively.

**Conclusion:**

Unspecific symptoms and hidden anatomic locations lead to delayed diagnosis and increased rates of metastatic dissemination. Distant metastasis is the main treatment failure in SNMM. Surgery with free margins remains the primary treatment for SNMM. Adjuvant radiotherapy might improve local control in individual cases but efficient systemic therapy is needed to improve outcome rates. To evaluate and define more effective targeted treatment options and improve outcome rates, homogeneous data and prospective multicentric analysis are needed.

## Introduction

Malignant mucosal melanoma of the nasal cavity and paranasal sinuses, is a rare disease entity with an incidence of 0.02–0.2 cases per 100,000 per year [1–6] and a 5-year survival outcome of approximately 30% [7, 8]. Despite technological advances and growing possibilities of treatment options for oncological patients in the last two decades, such as enhanced visibility for endoscopic surgery, 3‑dimensional radiotherapy or novel systemic therapies, local control and distant metastasis in patients with SNMM remain hard to handle and prognosis is poor [7, 9].

Macroscopically SNMM typically appear as a polypoid mass with or without pigmentation and the tumors are frequently ulcerated and may present in an unspecific variety of appearances, such as brownish, black, reddish, crimson, grey-white or even amelanotic imitating other tumors [10–12].

An SNMM typically has a delayed clinical presentation with unspecific and misleading symptoms, such as unilateral nasal obstruction and epistaxis, either occurring alone or combined [11, 14]. Given the unspecific clinical presentation and the variable macroscopic and microscopic appearances of SNMM, immunohistochemical investigations are often essential for diagnosis. A panel of markers including protein S‑100, HMB-45 and tyrosinase is recommended to accurately diagnose SNMM [12, 13].

Only about 5% of patients with SNMM have lymph node metastasis at the time of initial presentation. While only 10–15% of patients with SNMM initially present with distant metastasis, it has been shown that 40–70% of these patients develop distant metastases during the course of the disease, thus being the main treatment failure in SNMM [7, 15–17]. Most common sites of distant metastases are the lungs, liver, bone and brain [11, 15, 18].

The primary treatment of SNMM is surgical resection of the tumor. A wide resection to achieve clear margins should be the goal of every operation. In cases where free margins are obtained, patient outcome tends to be significantly better [19, 20].

In low grade tumor masses en bloc resection is possible, for larger tumors piecemeal resections were found to have similar oncological efficacy compared to en bloc resections by external surgical techniques [21–23].

Adjuvant treatment, such as radiotherapy and systemic therapy are frequently used; however, there are no standardized treatment regimens and recommendations mostly emphasize treatment on a patient to patient basis [24]. Conventional fractionation schedules consist of around 50 Gy delivered in 20 fractions of 2.5 Gy [25] and conventional chemotherapeutic agents, such as dacarbazine, carboplatin, cisplatin, vincristine, temozolomide and trofosfamide do not seem to have a satisfactory impact on regional and distant metastases [14, 25–27]. Novel targeted systemic therapies with monoclonal antibodies have already proven their effectiveness in cutaneous melanomas and have recently shown positive effects in mucosal melanomas [7, 28, 29]. For SNMM specifically, Zebrary et al. summarized reported frequencies of mutations in SNMM of five studies and found high variability between these studies: KIT 0–60%, NRAS 22–60% and BRAF 0–6% [30].

In this paper, we present our clinical experience with SNMM over a 19-year period at an academic center. The aim of our study was to expand the body of literature on this rare disease entity. In addition, we performed a literature review on SNMM to compare and discuss our results.

## Materials and methods

### Ethical considerations

The study was independently reviewed and approved by the local ethics committee of the Medical University of Graz and was performed in accordance with the ethical guidelines of the Declaration of Helsinki. Due to the retrospective nature of this study, patient informed consent was not obtained because clinical records were anonymized prior to analysis.

### Subjects

A retrospective chart review of all patients diagnosed and treated with SNMM from 2001 to 2019 at the Department of Otorhinolaryngology, Medical University of Graz, was performed. Patients were identified through the institutional head and neck tumor registry. Assessed clinical parameters were age at diagnosis, sex, symptoms at initial presentation, tumor localization, staging, metastasis, immunohistochemical markers and mutation status, treatment and survival.

Diagnosis of SNMM was confirmed histologically by experienced head and neck pathologists. Routine staging consisted of clinical examination (including nasal endoscopy) and imaging. Extension of primary lesion was assessed by paranasal magnetic resonance imaging (MRI) or computed tomography (CT). Presence of nodal and distant metastases was evaluated by neck/thoracic CT and abdominal ultrasonography or positron emission tomography CT. For staging the American Joint Committee on Cancer (AJCC) staging system for mucosal melanoma of the head and neck 7th edition, was used [31]*.* Treatment plan (surgery, radiotherapy, chemotherapy or combination therapy) was decided at the departmental interdisciplinary tumor board, based on tumor staging and patient’s clinical presentation. Patient follow-up consisted of a clinical examination including nasal endoscopy every 3 months and imaging (same modality as initial staging) every 6 months.

### Statistical analysis

SPSS© statistical software, version 25.0 (IBM, Armonk, NY, USA) was used for statistical analysis. Patient’s clinical characteristics were presented by descriptive statistics. The primary endpoint was the overall survival (OS). Survival curves were generated using the Kaplan-Meier method, including censodered data (= patient is still alive).

### Literature review

For the literature review, MEDLINE and PubMed central databases were searched with the terms “sinonasal” and “melanoma”. Studies with following inclusion criteria were used for further analysis: English or German language and full text available, patient number over 10, patient data not published since 1990, treatment including surgery and radiotherapy, and overall survival outcome data. A total of 302 primary matches were found and 279 were excluded on the basis of title and abstract. Of the studies 23 were taken into full-text assessment and 18 studies were chosen to undergo a narrative systematic review. A structured systematic review or meta-analysis with quantitative comparison outcomes such as survival, treatment modalities, surgery alone, surgery and radiotherapy, surgery and chemoradiotherapy and staging, could not be performed because the available data are not comparable to each other due to heterogeneity of data in the studies.

## Results

### Demographics

Overall, 12 patients were diagnosed with SNMM from 2001 to 2019 at the Department of Otorhinolaryngology, Medical University of Graz. Mean age of all patients was 66.5 years (σ = 17.4), median age was 70 years (range 43–88 years). There were 6 (50%) female and 6 male (50%) patients (Table 1).

**Table 1:** Patient’s clinical characteristics of the present study

Characteristic	Number of patients (%)
Total Number of patients	12 (100)
*Sex*	
Male	6 (50)
Female	6 (50)
*Age (years)*	
<65	6 (50)
>65	6 (50)
*Location*	
Nasal cavity^a^	7 (58)
Paranasal sinuses^b^	2 (17)
Both	3 (25)
*Principle symptoms*	
Epistaxis + nasal obstruction	7 (58)
Epistaxis only	2 (17)
Nasal obstruction only	1 (8)
Nasal/orbital pressure	1 (8)
Collapse	1 (8)
*Staging*	
T3	7 (50)
T4a	2 (25)
T4b	3 (25)
*Treatment*	
Surgery only	2 (17)
Surgery + adjuvant radiotherapy	3 (25)
Surgery + adjuvant chemoradiotherapy	3 (25)
Surgery + immunotherapy	1 (8)
Radiotherapy + chemotherapy	1 (8)
Radiotherapy alone	1 (8)
Chemotherapy alone	1 (8)

^a^Nasal cavity includes: nasal vestibule and atrium, nasal septum, nasal conchae and sphenoethmoidal recess

^b^Paranasal sinuses include: maxillary sinus, ethmoidal sinus, frontal sinus, sphenoidal sinus

### Localization

In 6 cases (50%) the primary tumor location was the nasal cavity, in 1 case (8%) the paranasal sinuses and in 4 cases (34%) both the nasal cavity and the paranasal sinuses. In 1 patient (8%) the primary tumor site was the nasal septum and ethmoid bone with infiltration of the skull base (Table 2).

**Table 2:** Clinical parameters of each patient included in the present study

No	Age (years)	Sex	Location	Staging	Surgery	RT	ST	ND	OS
1	54	F	NC	T3N0M0	FESS	Yes	HDI, CBDCA	No	24
2	41	M	ES, SS	T3N0M0	FESS	Yes	CBDCA	No	104.1
3	81	F	NC, NS, SER	T4aN0M0	FESS; revisional FESS	Yes	No	No	9.2
4	83	F	NC, MS, ES, FS	T4aN0M1	No surgery	Yes	DTIC	No	19.3
5	80	F	NC, NS, SER	T3N1M0	FESS	Yes	No	No	11.5
6	79	M	NC, MS, SB, Ob, HP	T4bN0M0	No surgery	Yes	No	No	22.1
7	82	F	NC, MS	T3NXM0	FESS	No	No	No	8.4
8	59	F	NC	T3NXM0	FESS; revisional OR, FESS	No	No	Yes	88+
9	47	M	NC	T3N0M0	FESS	Yes	No	Yes + LN	55.8+
10	61	M	NC	T3NXM0	FESS	Yes	DTIC	Yes	14.2
11	88	M	NC, FS, Ob	T4bN0M1	No surgery	No	Ixoten	No	7.1
12	43	M	NS, EB, SB	T4bN0M1	FESS	No	Nivolumab + Ipilimumab	No	9

*NC* nasal cavity, *NS* nasal septum, *SER* sphenoethmoidal recess, *MS* maxillary sinus, *ES* ethmoidal sinus, *EB* ethmoidal bone, *SS* sphenoid sinus, *FS* frontal sinus, *Ob* infiltration of orbit, *SB* skull base, *HP* hard palate, *FESS* functional endoscopic sinus surgery, *OR* open resection, *RT* radiotherapy, *ST* systemic therapy, *HDI* high dose interleukin, *CBDCA* carboplatin, *DTIC* dacarbazine, *ND* neck dissection, *LN* submandibular lymph node, *OS* overall survival (months)

### Symptoms

In 7 cases (58.3%) primary symptoms were epistaxis and nasal obstruction, in 2 cases (16.6%) nasal obstruction was the only symptom, 1 patient (8.3%) had epistaxis only, 1 patient (8.3%) had a feeling of pressure in the nasal cavity and left orbit, and 1 patient (8.3%) had a collapse due to severe metastatic progression of the principal tumor (Table 1).

### Staging

The AJCC 7th edition staging system for mucosal melanoma of the head and neck was used in this series. At the time of diagnosis 7 patients (58.3%) were staged T3, 2 patients (16.6%) T4a and 3 patients (25%) T4b (Table 2).

### Immunohistochemical markers and mutation status

In 6 cases (50%) melan A, protein S‑100 and HMB-45 showed positive immunohistochemical reactions. Melan A showed positive immunohistochemical reactions in 9 cases (75%), protein S‑100 in 8 (66.6%), HMB-45 in 6 cases (50%) and tyrosinase and vimentin, each in 1 case (8.3%). In 2 cases (16.6%) specific information was unavailable because diagnosis was made in other institutions and in 1 case (8.3%) the immunohistochemical activity was not assessed. To find out whether targeted treatment options are applicable, mutation status of BRAF, KIT and NRAS was assessed in several cases; however, in all cases assessed, genetic analysis only brought up wild-type sequences and did not show mutant phenotypes (Table 3).

**Table 3:** Immunohistochemical markers and mutation status of all included patients

Patient	Immunohistochemical reaction	BRAF	KIT	NRAS
1	Na	Na	Na	Na
2	Melan A, S‑100	WT	WT	Na
3	Melan A, S‑100, HMB-45	Na	Na	Na
4	Melan A, S‑100	Na	WT	Na
5	Melan A, S‑100, HMB-45	WT	WT	Na
6	Not available	WT	WT	Na
7	Melan A, HMB-45	Na	WT	Na
8	Melan A, S‑100, HMB-45, Tyrosinase	Na	Na	Na
9	Melan A, S‑100, HMB-45	WT	WT	Na
10	Melan A, S‑100, HMB-45, Vimentin	WT	WT	WT
11	Not available	WT	WT	WT
12	Melan A, S‑100, HMB-45	WT	WT	WT

*Na* not assessed, *WT* wild-type

### Treatment

Out of 12 patients 9 (75%) underwent endoscopic surgery. Of those, 2 patients had revision surgery due to local recurrence, of which 1 patient had an open resection via midfacial degloving because of local destructive growth of the tumor. In 3 patients, a functional neck dissection was performed. Of those patients who had undergone surgery, 6 patients received adjuvant treatment: 3 patients received radiotherapy, 3 received radiotherapy plus chemotherapy. 1 surgically treated patient received postoperative immunotherapy (nivolumab + ipilimumab). 2 patients underwent surgery alone, 3 patients who did not undergo surgery received radiotherapy plus chemotherapy (8.3%), radiotherapy alone (8.3%) and chemotherapy alone (8.3%), respectively. Chemotherapeutic agents used were dacarbazine, carboplatin and ixoten.

### Metastasis, locoregional

At the time of diagnosis 1 patient (8.3%) had afflicted supraclavicular, perihepatic and perirenal lymph nodes, 3 patients (25%) developed cervical lymph nodes metastases 2, 6 and 39 months after surgery.

### Metastasis, distant

At the time of diagnosis, 1 patient (8.3%) had liver metastasis, 1 patient (8.3%) had metastases in lungs and liver, and 1 patient (8.3%) had distant metastases in lungs and pleura, the adrenal glands, pancreas and peritoneum, 4 patients (33.3%) developed distant metastasis 2, 10, 11 and 16 months after initial treatment. Affected organs were lungs in 4 cases, liver in 3 cases, retroperitoneal soft tissue in 2 cases, kidneys in 2 cases and in 2 cases bony structures in the thoracic and lumbar spinal column and the femur.

### Survival

**Fig. 1:**
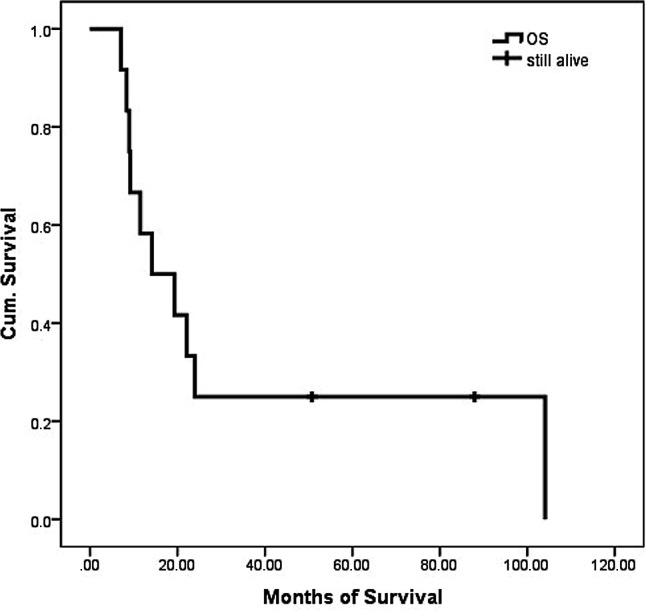
Overall survival (OS) of all included patients

**Fig. 2:**
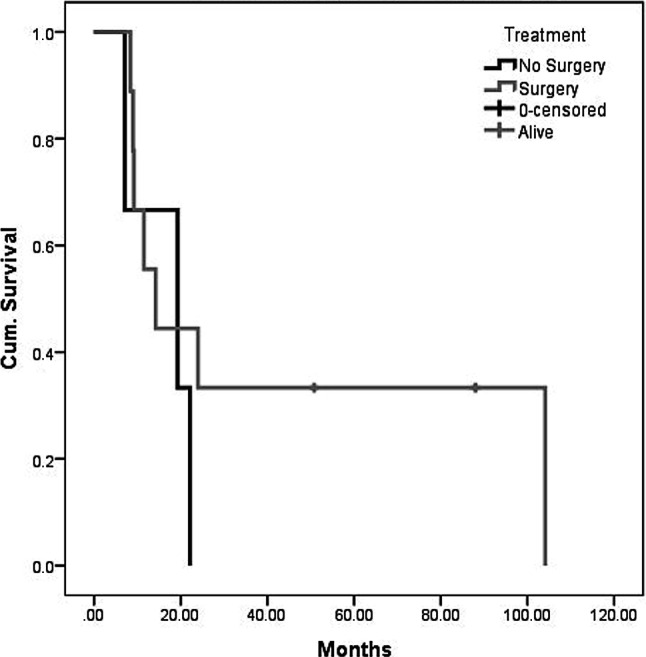
Overall survival of patients with and without surgery

**Fig. 3:**
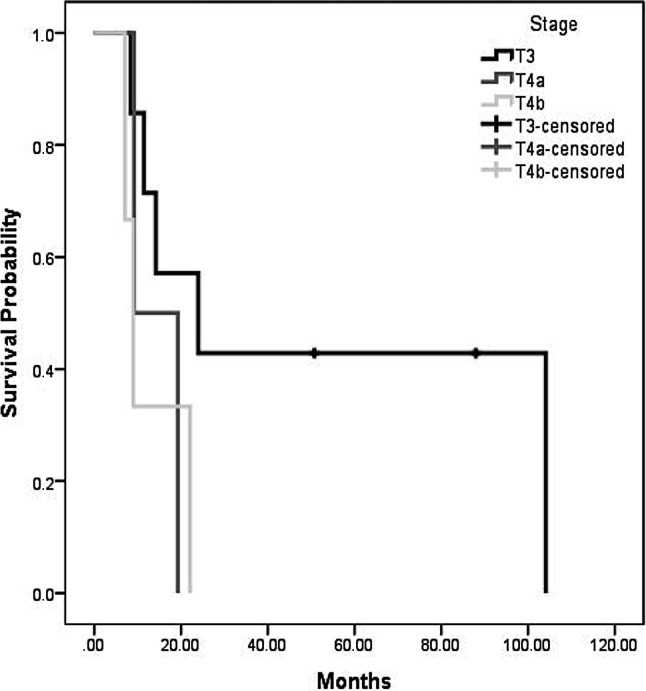
Overall survival stratified for tumor stage

As per April 2019, 2 patients are still alive at 88 (FESS + open revision via midfacial degloving + neck dissection + revisional FESS) and 51 (FESS + radiotherapy + neck dissection and resection of functional lymph nodes) months, respectively. For all patients, the median OS was 16.7 months with a range from 7.1 to 104.1 months, while mean survival was 30.6 months (Fig. 1). The 1‑year OS was 58.3%, 3‑year OS was 25%, 5‑year OS was 18.2%. For patients who underwent surgical resection, median OS was 14.2 months with a range from 7.1–104.1 months, while mean survival was 35.4 months, and 1‑year OS, 3‑year OS and 5‑year OS were 55.6%, 33.3%, and 22.2%, respectively (Fig. 2). For 3 patients, who did not receive surgery, the mean OS was 16.7 months, with 19, 22 and 7 months, respectively. Stratified by tumor stage, mean overall survival was 52.9, 14.3 and 12.7 months for T3, T4a and T4b, respectively (Fig. 3).

### Literature review

The results of the literature review are listed in Table 4, including 18 articles and the results of the present series. Konuthula et al. [3] and Ajmani et al. [32], as well as Moreno et al. [14] and Amit et al. [24], each had overlapping sources of patient data from the National Cancer Data Base (NCDB) and the MD Anderson Cancer Center.

**Table 4:** Literature review

Author	Data	Patients *n*	Mean age (years)	Gender *n* (%)	Location *n* (%)	Staging *n* (%)	Treatment *n* (%)	Surgical approach	Margin status *n* (%)	5‑year OS (%)
Khademi et al. 2011 [33]	1995–2005	18	65	M: 16 F: 2	NC: (62) ES: (19) MS: (19)	B: I: 8 II: 7 III: 3	S + RT: 18 CT: 5	n/a	n/a	23
Konuthula et al. 2017^a^ [3]	2004–2010	695	69	M: 316 F: 379	NC: 470 PNS: 225	n/a	S: 206 S + RT: 271 S + CRT: 49 S + CT:29 RT: 42 CT: 21	n/a	Neg: 300 Pos: 127 UK: 268	21.7
Lombardi et al. 2016 [19]	2003–2012	58	71	M: 21 F: 37	NEC: 51 MS: 6 FS: 1	A7: T3: 30 T4a: 17 T4b: 11	S:42 S + RT: 13 S + CRT: 2 S + CT: 1	OR: 7 ER: 47 Both: 4	Neg: 41 (71) Pos: 17 (29)	29
Martin et al. 2004 [18]	1991–2002	20	77	M: 8 F: 12	NC: 8 PNS:3 NC + PNS: 9	A6: T1: 3 T2: 6 T3: 3 T4: 8	S: 2 S + RT: 15 S + CT: 1 RT: 2	n/a	n/a	2YOS: 23
Meng et al. 2014 [34]	2000–2010	69	66	M: 37 F: 32	NC: 36 LNW: 19 MS: 21 ES: 18 NS: 4 SS: 4	CA7: III: 37 IVA: 27 IVB: 5	S: 27 S + RT: 24 S + RT + CT: 18	OR: 41 ER: 28	n/a	21.8
Moreno et al. 2010^b^ [14]	1993–2004	58	63	M: 35 F: 23	LNW: 25 NS: 14 MS:12 ES: 5 SS: 1 NPX: 1	A6: T1: 16 T2: 19 T3: 12 T4: 11	S: 25 S + RT: 31 RT: 2 Adj CT: 14 Adj IT:22	OR: 46 ER: 10	Neg: 46 Pos: 12	38.7
Narasimhan et al. 2009 [35]	1995–2007	18	68	M: 8 F:10	NC: 6 NS: 3 MS: 12	A6: I: 2 II: 2 III: 4 IV: 10	S: 18 Adj RT: 10 Adj CT: 10 Adj IT: 8	n/a	n/a	34
Roth et al. 2010 [36]	1992–2007	25	71	M:8 F:17	NC: 11 NS: 4 MS:5 ES: 5	n/a	S: 11 S + RT: 7 S + CRT: 2 S + CT:1 RT: 3 NoT: 1	OR: 6 ER: 15	Neg: 16 Pos: 5	33
Samstein et al. 2016 [37]	1998–2013	78	68	M: 38 (49) F:40 (51)	NC: 52 PNS: 26	A7: T3: 39 T4a: 29 T4b: 8 UK: 2	S: 14 S + RT: 58 S + CRT: 6	n/a	Neg: 30 Pos: 24 UK: 24	31
Swegal et al. 2014 [38]	1998–2012	25	67	M: 14 F:11	NC: 17 PNS: 8	CA7: III: 9 IVA: 6 IVB: 8 IVC: 2	S: 3 S + RT: 22 Adj Sys: 6	OR: 13 (52) ER: 12 (48)	Neg: 14 (56)	2YOS: OR: 64% ER: 44%
Tajudeen et al. 2014 [39]	1991–2011	14	64	M: 7 F: 7	NC: 11 PNS: 3	A6: T1: 6 T2: 2 T3: 0 T4a: 6	S: 3 S + RT: 8 S + CRT: 2 S + UK: 1	n/a	Neg: 10 (71%) Pos: 4 (29%)	35
Vandenhende et al. 2012 [40]	1991–2008	25	68	M:12 F: 13	LNW: 11 MS: 4 NS: 5 NF:1 Other: 4	A7: T3: 6 T4a: 8 T4b:11 N1: 1	S: 80 S + RT: 15 RT: 1 Pal: 1	OR: 12 ER: 11	Neg: 20 Pos: 5	3YOS: T3: 100 T4: 52
Won et al. 2015 [9]	1994–2013	155	63	M: 81 F: 74	NC: 99 NS:54 MS:34 ES:28 FS: 6 SS: 6 SB: 6 Orbit: 9 NPX: 5 NLC: 3 Skin: 6	CA7: III: 67 IVA: 65 IVB: 9 IVC: 14	S: 48 S + RT: 54 S + Sys: 28 S + CRT: 11 UK: 14	OR: 63 incl ER:70	n/a	40.1
Yu et al. 2015 [41]	1999–2013	29	62	M:18 F:11	LNW: 12 NS: 7 MS:5 ES: 5	A6: T1: (34) T2: (34) T3: (21) T4: (17)	S: 22 RT: 28 CT: 17	EA: 7 ER: 15	n/a	27.6
Huang et al. 2007 [5]	1994–2005	15	69	M: 8 F:7	n/a	B: I: 14 II: 1	S + RT: 10 S + CRT: 3 CRT: 1 UK: 1	OR: 5 ER: 8	n/a	33
Ajmani et al. 2017^a^ [32]	2004–2013	696	n/a	n/a	NC: (74.6) PNS: (25.4)	CA7: III: (49.5) IVA: (39.1) IVB: (11.4) UK: (24.3)	S: 305 S + RT: 399 RT incl adj CT	n/a	Neg: (73)	24 S: 24.3 S + RT: 8.2
Amit et al. 2017^b^ [24]	1991–2016	152	64	M: 65 F: 87	NC: 119 PNS: 32 UK: 1	A7: T3: 97 T4a: 54 T4b: 3	S: 57 S + RT: 73 S + CRT:8 S + RT + NCT: 14	n/a	n/a	41 S:39 S + RT: 41 S + CRT: 47 S + RT + NCT: 27
Gal et al. 2011 [6]	2000–2007	304	n/a	M:133 F:171	NC: 199 MS: 46 ES:27 ACCS:32	CA7: III: 98 IVA: 77 IVB: 34 IVC:37 UK:58	S: 128 S + RT: 120 RT: 23 UK: 33	n/a	n/a	24.2
Present	2001–2019	12	67	M:6 F:6	NC: 7 PNS: 2 UK: 3	CA7: III: 6 IVA:2 IVB: 1 IVC: 3	S: 2 S + RT: 3 S + CRT: 3 S + IT: 1 Pal: 3	ER: 8 Incl ER: 1	n/a	18.2

*NC* nasal cavity, *NS* nasal septum, *NV* nasal vestibule, *NF* nasal fossa, *NEC* nasoethmoidal complex, *LNW* lateral nasal wall, *MS* maxillary sinus, *ES* ethmoidal sinus, *FS* frontal sinus, *SS* sphenoid sinus, *ACCS* accessory sinus, *PNS* paranasal sinuses, *NPX* nasopharynx, *NLC* nasolacrimal duct, *SB* skull base, *UK* unknown, *B* ballantyne staging system, *A6* AJCC 6th edition staging of primary tumor, *A7* AJCC 7th edition staging of primary tumor, *CA7* AJCC 7th edition clinical staging, *S* surgery, *RT* radiotherapy, *CRT* chemoradiotherapy, *CT* chemotherapy, *IT* immunotherapy, *Sys* systemic therapy not otherwise specified, *NCT* neoadjuvant chemotherapy, *Pal* palliative therapy, *OR* open resection, *ER* endoscopic resection, *EA* endoscopic assisted resection, *OS* overall survival, *n/a* not available/not applicable

^a^Overlapping source of patient data: National Cancer Data Base

^b^Overlapping source of patient data: MD Anderson Cancer Center

## Discussion

Sinonasal mucosal melanoma is a rare tumour entity with an average 5‑year OS rate not exceeding 35%. Given the rarity of this tumor, its anatomically difficult location und its histopathological and immunohistochemical peculiarities, survival rates remain poor compared to cutaneous melanomas. Although some prognostic findings were made by several single center and multicentric studies, nationwide database reviews and meta-analyses, there is still no consensus for a standard of treatment regarding adjuvant therapy. The reasons for this might be the retrospective character of existing studies and their diversity in terms of patient selection, staging and treatment-specific survival outcome, which makes bias-free comparison and analysis difficult.

While smaller, single center case series have a limited number of cases and therefore have no specific inclusion criteria, multicentric and nationwide studies can afford more detailed inclusion criteria in terms of patient history, initial staging, curative and palliative treatment intent. Given these differences, statistical comparison and analysis would increase the probability of selection bias and decrease its scientific value.

Due to the anatomical location of the tumors, the lack of symptoms in lower stages might contribute to delayed diagnosis in advanced stages and the generally poor outcome rates.

The vast majority of patients throughout the literature had either nasal obstruction or epistaxis or both as principle symptoms [5, 9, 14, 18, 19, 33–36, 40, 41], which is in accordance with our patient series.

Documentation of involved structures varied widely in reviewed studies, subdivisions ranging from nasal cavity and paranasal sinuses to structures of the nasal cavity. Some authors divided roughly into nasal cavity and paranasal sinuses for primary origin while others divided particularly into specific anatomic structures of the nasal cavity such as the lateral nasal wall or the nasal septum, or each one of the sinuses. Also, as bigger tumors involved more than one site, some authors list more than one location for one tumor.

Numerous prognostic factors for SNMM were found in the literature. Patients with primary tumors arising from the nasal cavity had better survival outcome than those originating from any of the paranasal sinuses [3, 9, 14, 24, 36, 37, 39]. A possible cause for this divergence could be that paranasal sinus lesions might be diagnosed at a more advanced tumor stage than lesions in the nasal cavity due to their hidden anatomic location [2, 3, 6, 42]. Furthermore, negative margins after resection [3, 19, 32, 43], advanced stage [6, 18, 19, 33, 37] and, interestingly, the level of pigmentation [14, 41] turned out to be significant prognostic factors.

There are still several different methods at use to stage SNMM which makes comparison of different series more difficult. Although some authors argued that the former TNM system of 2002 (AJCC 6th) [44] had adequate prognostic value and was better known internationally [18], several studies have shown that the accuracy of the newer AJCC 7th edition staging system is equal or superior to others, especially in the staging of advanced tumors [4, 6, 45]. No significant changes regarding mucosal melanomas were made in the AJCC 8th edition staging system.

Complete tumor excision is commonly accepted as standard treatment for patients with SNMM. Several studies have shown that survival is significantly better in patients with free surgical margins [3, 19, 43]. Due to the complex anatomy of the sinonasal cavities near vital structures, and the tumor patterns of locally invasive and destructive growth, surgical resection with free margins is challenging and may not be possible in many cases [14, 34, 37, 40]. Moreover, radical surgical procedures which often come with significant cosmetic and functional deficiencies do not seem to be justified when over 40% of the patients develop distant metastasis after achieving local control with surgery [14, 22, 24, 46]. Because of the lack of prospective, randomized trials concerning SNMM it is not possible to collect data about the quality of life of patients who underwent different surgical approaches [47]. Most studies show similar outcomes in comparing open to endoscopic surgery. Amit et al. showed that the oncological efficacy of endoscopic surgery was similar to open surgery but with a potentially lower risk of morbidity [24].

Won et al. and Moreno et al. demonstrated significantly higher survival rates in patients who underwent an endoscopic resection; however, since an external approach tends to be used in higher staged tumors, the possibility of a selection bias cannot be excluded in these studies [9, 14]. Nevertheless, external or combined (endoscopic and external) approaches are still recommended as effective surgical options in SNMM massively infiltrating surrounding and bony structures [19, 23].

There is broad consistency in pointing out that adjuvant radiotherapy improves local control in reducing local tumor recurrence rate but has no significant effect on survival outcome [3, 9, 14, 16, 33, 36, 37, 40, 41, 48, 49]. Information about radiation dose, fractionation and techniques is inconsistent and different radiation regimens have been presented. Moreno et al. found improvement of locoregional control when a total dose of more than 54 Gy was used in a standard fractionation schedule [14], which was confirmed by Yu et al. and Wada et al. [41, 49]. Meng et al. and Caspers et al. found improvement of local control giving mean total dosages of 63.4 Gy and 64 Gy, respectively [34, 50]. An even higher dosage above 70 Gy was suggested by Greenwalt et al. to increase locoregional control [51].

In a recently published retrospective study of 152 patients treated at the MD Anderson Cancer Center, Houston, Texas, Amit el al. described a standard scheme for radiation therapy using intensity modulated radiation therapy with a total dose of 60–70 Gy at 1.8–2 Gy per fraction over 6–7 weeks [24]. Because of the heterogeneity of various studies, it is not always clear which was the prevailing argument for the use of higher dose fractions and since hypofractionation is commonly used in palliative cases, a selection bias cannot be excluded [25]. In a recent nationwide study by Ajmani et al. the addition of standard adjuvant therapy (RT and CRT) does not seem to offer a survival benefit except for advanced tumors staged IVB. They conclude, the same as other authors that due to the lack of survival benefit and the added morbidity of radiation, radiotherapy should be prescribed individually and with caution [7, 32].

Systemic therapy in SNMM does not seem to have the desired effects on survival outcome in advanced stages of the disease [14, 16, 25, 48]. While regional lymph metastases are uncommon, distant metastases are one of the main treatment failures of SNMM. Classical chemotherapeutic agents like dacarbazine, or cisplatin derivatives do not seem to significantly impact SNMM, while other treatment approaches like novel immunotherapeutic agents indicate better response rates and disease control rates for mucosal melanomas [14, 25, 52]. Due to the rarity of SNMM, most studies regarding novel systemic therapies cover subgroups of melanoma including cutaneous and mucosal melanomas.

Studies regarding the effectiveness of adjuvant biochemotherapy indicate that the addition of interleukin 2 and interferon alpha‑2 to chemotherapeutic agents like dacarbazine or carboplatin may not improve durable responses or survival outcome [3, 53, 54]. Gene expressions in mucosal melanomas like *c‑KIT*, *NRAS* or *BRAF* might be of potential use for selective inhibitors. Although mutations of these gene expressions were found by other authors, only wild-type sequences were found in tumors assessed in the present series. In a study by Hodi et al. patients with *c‑KIT* mutations in advanced disease, were treated with the tyrosine kinase inhibitor imatinib and had a tumor response rate of 54% and an overall disease control rate of 77% [55]. Nivolumab and ipilimumab are immune checkpoint inhibitors. Nivolumab showed improved overall survival and better response rate versus dacarbazine in patients with *BRAF* wild-type melanoma in a phase III study by Robert et al. [56]. The combination of nivolumab and ipilimumab in patients with mucosal melanoma showed higher efficacy than either agent alone [28].

## Conclusion

Surgery with confirmed free margins remains the standard treatment for SNMM. With technological advances in terms of visualization and instruments, endoscopic resections do not seem to be inferior to external approaches; however, the surgical approach ought to be chosen based on the probability to gain free margins. Adjuvant radiotherapy with a total radiation dose of 54 Gy or higher with standard fractionation schemes might be considered if margin status cannot be assessed with certainty or complex anatomic circumstances of the primary tumor make a definite assertion difficult. Local recurrence and distant metastasis remain the main treatment failures in SNMM, even after achieving local control and R0 resections. Although standard chemotherapy does not seem to have a satisfactory impact on SNMM, newer biological systemic agents like imatinib or the combination of ipilimumab plus nivolumab might improve overall survival of this fatal tumor. For further evaluation of effectiveness of these novel therapies it is important to consider the possibility of different genetic alterations between the tumor cells of cutaneous melanoma, mucosal melanoma, and particularly sinonasal mucosal melanoma. Therefore, it is necessary to investigate treatment modalities and outcomes distinctly for SNMM.

The number of different heterogeneic single-centered or multi-centered case series and nationwide studies, all with a retrospective character, make reasonable comparison with useful statements regarding treatment options, including systemic therapy and survival outcome in SNMM difficult. Many authors in literature conclude similarly that prospective multicentric studies are needed to reach higher patient numbers and improve scientific conclusions. Beswick et al. designed a web-based multi-institutional registry for patients with sinonasal malignancies, a web-based, secure database to prospectively collect data in cases diagnosed with sinonasal malignancies [57]. This could be an incitement for further research of similar portals, especially in Europe.

In conclusion, early diagnosis, free surgical margins and effective systemic therapy are needed to improve survival outcome in SNMM.
